# Exploring the gap between refugee-background communities’ needs and existing community-based physical activity programs in Australia

**DOI:** 10.1093/heapro/daad039

**Published:** 2023-05-15

**Authors:** Georgia Hawkins, Christina Malatzky, Susan Wilson, Kaeleen Dingle

**Affiliations:** School of Public Health and Social Work, Queensland University of Technology, Kelvin Grove 4059, Australia; School of Public Health and Social Work, Queensland University of Technology, Kelvin Grove 4059, Australia; School of Public Health and Social Work, Queensland University of Technology, Kelvin Grove 4059, Australia; School of Public Health and Social Work, Queensland University of Technology, Kelvin Grove 4059, Australia

**Keywords:** refugee-background communities, physical activity, cultural safety, collaboration, capacity building

## Abstract

Physical activity programs run by local government, public health and not-for-profit sectors are a key public health strategy for improving rates of physical activity within local communities. However, these programs are underutilized. This is especially the case among members of refugee-background communities whose participation could have far-ranging and multilevel benefits. To explore how greater engagement among refugee-background communities with these programs could be fostered in Brisbane, Queensland, Australia, a qualitative study was undertaken from the perspectives of both community-based physical activity program providers and agencies involved in delivering services to refugee-background communities. This study involved a series of semi-structured interviews with a purposive sample of personnel from agencies that work with individuals and families from refugee-background communities and organizations that provide low-cost or no-cost physical activity programs and initiatives. Reflexive thematic analysis was used to interpret meaning from these data. Three themes relating to how participation in community-based physical activity programs could be improved among refugee-background communities were identified: improving cultural safety through intersectoral collaboration; confronting constraints imposed by the broader public health policy environment; and building capacity and empowering the community to diversify the sector. The findings highlight the importance of localized, deep-level intersectoral collaborations in bridging the gap between the health and social care needs of refugee-background communities and existing physical activity programs. However, a range of systems-produced barriers to the creation of such collaborations must be addressed to enable local actors to help mitigate and address the systemic exclusion of marginalized populations from participation in broader society.

## INTRODUCTION

Physical activity is key to improving health and well-being throughout the lifecourse (Wheeler *et al.*, 2018; Jeon *et al.*, 2019; Hargreaves *et al.*, 2021). Being physically active can help to prevent many serious chronic illnesses, such as Type 2 diabetes, cardiovascular disease and cancer (Rhodes *et al.*, 2017; Australian Institute of Health and Welfare, 2020), which are the leading causes of death and disability globally (Institute for Health Metrics and Evaluation, 2018; Lin *et al.*, 2020). Many other benefits of physical activity for physical health, including improved mobility and bone health (Rodriguez-Gomez *et al.*, 2018; Arnett *et al.*, 2019), are well established in the literature. Engagement in physical activity also has a positive effect on mental health (Kim *et al.*, 2012; Peddie *et al.*, 2020; Montague and Haith-Cooper, 2022) and emotional and social well-being (Pasanen *et al.*, 2014; Litwiller *et al.*, 2017). Yet rates of physical activity are declining, and sedentary time is increasing in many countries in the global North and increasingly in the South (Eaglehouse *et al.*, 2017; Holtermann *et al.*, 2017; Lai *et al.*, 2019). The economic and social costs of these trends are substantial for global society (Oldridge, 2008; Cunningham *et al.*, 2021). Thus, increasing rates of physical activity within populations is a key public health objective. In many countries, physical activity programs run by local government, public health and the not-for-profit sectors have been established to assist with this objective at the community level (Cunningham *et al*., 2021; Lee and Ho, 2021).

The minimal to no cost for participation in community-based physical activity programs makes them financially accessible to a broad range of people. However, what determines accessibility in a broader sense is complex and multi-faceted (Levesque *et al.*, 2013). Currently, while potentially increasing physical activity across a local community, these programs are underutilized and, in many cases, unsustainable (Ho *et al.*, 2019). In the current socio-political environment, short-term funding cycles have become the norm. This creates greater uncertainty about the longevity of many health promotion initiatives (Scheirer and Dearing, 2011; Schell *et al.*, 2013; Smith *et al.*, 2019). Further, those in the community who would likely most benefit from participation in community-based physical activity programs are the least likely to engage (Russell *et al.*, 2013; Halliday *et al.*, 2014). Most program participants are already comparably rich in the resources needed to support good health (Russell *et al*., 2013).

Health and social care needs are high across refugee-background communities (Ross Perfetti *et al*., 2019; Rogers *et al.*, 2020; Shrestha-Ranjit *et al.*, 2020; Hawkes *et al.*, 2021). In Australia, levels of participation in physical activity are lower within refugee-background communities when compared to the broader population (Caperchione *et al.*, 2009; Caperchione *et al.*, 2011; Russell *et al*., 2013; Ball *et al.*, 2015). In Queensland, a recent cross-sectional study that examined physical activity among culturally and linguistically diverse communities found that only one-third of respondents met current health guidelines for physical activity, which compares to just over half within the general Australian population (Gallegos *et al*., 2020). Yet participation in physical activity plays a particularly important role in fostering a sense of social connectedness and belonging among refugee-background communities (Spaaij, 2012; Spaaij, 2015; Mohammadi, 2019). Refugee-background community members often seek opportunities to form friendships and participate in broader community activities (Dandy and Pe-Pua, 2015; Bourke *et al.*, 2019). Conversely, direct personal contact and friendship with cultural others can reduce racial prejudice among those belonging to culturally dominant groups within a particular society (McLaren, 2003; Dandy and Pe-Pua, 2015). Thus, increasing the participation of refugee-background communities in community-based physical activity programs is likely to have positive, multilevel effects on the health of broad publics.

A developing area of research is how to better engage refugee-background communities in community-based physical activity programs. In the Australian context, research has sought to investigate why refugee-background communities tend not to engage with these programs. This research has identified a range of barriers to such participation, including a lack of broader community acceptance of different cultures and cultural practices; cultural perceptions of health and illness; experiences of trauma; language; religion; lack of social support; and socioeconomic constraints. In addition, environmental factors such as concerns about safety among women living in ‘high crime’ and violence areas, lack of familiarity with the local neighbourhoods and change in climate, especially when relocating from consistently warm and dry climatic zones into those with more variable conditions are recognized as barriers (Caperchione *et al*., 2009; Caperchione *et al*., 2011; Cyril *et al.*, 2016; Rosso and McGrath, 2016; Hartley *et al.*, 2017). Cyril et al. (Cyril *et al*., 2016) and Rosso and McGrath (Rosso and McGrath, 2016) found that service providers typically hold insufficient knowledge about these barriers.

Contemporary literature has also explored what enables refugee-background communities to participate in physical activity initiatives. Enablers include delivering physical activities that benefit and interest the target group, designing low/no-cost programs and locating programs near refugee-background communities to reduce transport barriers (Caperchione *et al*., 2009; Mohammadi, 2019). Importantly, existing literature has identified communication between physical activity program providers and stakeholders who engage with refugee-background communities, particularly to overcome cultural and language barriers, as a key enabler for participation (Mohammadi, 2019). In a scoping review of intersectoral approaches to attaining the right to health for refugee-background communities, Ho et al. (Ho *et al*., 2019) argue that stronger collaboration across the health and social care system is needed to enhance awareness about and the accessibility of existing programs for supporting good health. However, to date, there has been limited investigation into how this could be achieved within contemporary community health sectors that may be underutilized or siloed. This project was designed to address the gaps in knowledge about how greater engagement among refugee-background communities with these programs could be fostered from the perspectives of both community-based physical activity program providers and agencies involved in delivering services specifically to refugee-background communities.

## METHODS

This study employed a qualitative research design to explore service providers’ perspectives on the barriers to and enablers of refugee-background communities’ participation in community-based physical activity programs in Brisbane, Queensland, Australia, where there is a renewed focus, evidenced through the Activate! Queensland Strategy: 2019–29 (State of Queensland, 2019), on addressing barriers to physical activity, especially among groups who experience greater vulnerability. Specific attention was given to how, from the participants’ perspectives, the service sector could improve participation among members of refugee-background communities. Qualitative research approaches enable the exploration of social systems and structures that maintain social marginalization and health inequities and can assist with developing and implementing initiatives that seek to redress these injustices (Peter, 2015). Qualitative methods provide researchers with in-depth insight into the processes and structures that influence decision-making (Broom and Willis, 2007), which Pope and Mays (Pope and Mays, 1995) argue is critical to examining the provision of health services and programs.

An environmental scan was undertaken to identify service delivery agencies that work with adults from refugee-background communities (participant Group 1) and organizations that provide low-cost or no-cost physical activity programs and initiatives (participant Group 2) in Brisbane that could participate in an individual or small group interview as part of this research (Graham *et al.*, 2008). This involved conducting internet searches and utilizing online sources (e.g. service provider websites) as well as initiating telephone conversations and email correspondence to ensure the scan was comprehensive and identifying the most appropriate contact person (e.g. program coordinator) at each service (Rowel *et al.*, 2005). The environmental scan identified 34 potential Group 1 and 12 potential Group 2 participants. All potential participants were contacted via telephone or email correspondence and provided with an information sheet. This sheet outlined the purpose of the study and that participation entailed taking part in either an individual or small group (in the circumstance that multiple personnel were involved in service delivery in the one organization, and the preference of all was to be interviewed together) interview either in person (when possible under COVID-19 restrictions) or virtually with the first author. The information sheet made it clear that not participating in the research would have no impact on current or future relationships with Queensland University of Technology.

Semi-structured interview guides were used to facilitate discussion addressing the identified research aims. Interviews were digitally recorded, and the interviewer also took handwritten notes during each interview and kept a record of their personal reflections and emergent ideas based on interview discussions related to the research aims. This approach helped document the interviewer’s understanding of data, including possible connections between ideas interpreted from the data, which informed later analytical work (Jasper, 2005).

The analysis of interview data was guided by Braun and Clarke (Braun and Clarke, 2013, Braun and Clarke, 2021) six-phase iterative analytical process for undertaking reflexive thematic analysis. It involved: (i) becoming familiar with the data through a process of active listening and re-listening before transcribing segments of the data identified as relevant to the research aims; (ii) generating codes in an inductive, recursive manner to all segments of data across the data set that held some relevance to the research aims; (iii) generating themes across the data set by reviewing codes, examining how these codes related to one another and identifying the concepts or ideas that explained these connections; (iv) reviewing themes to ensure there was a logical pattern/relationship between codes that underpinned each proposed theme and that the interpretation of data articulated in the identified theme was useful in relation to the research aims posed at the outset; (v) defining and naming the themes related to the research questions and organizing data extracts from across the data set under these themes; and (vi) producing the write-up of findings (presented below). The first author led this process. The second author engaged in their own Phase 1 process, sense-checked the first author’s coding in Phase 2 and developed the interpretation of data in Phases 3–6 (Byrne, 2021). The third and fourth authors similarly worked collaboratively to enrich the meanings interpreted from the data. Throughout this process, care was taken to understand and be conscious of the contexts and positionings of participants (see Malatzky *et al.*, 2020a) and, in the write-up, to be mindful of how participants are (re)presented (Finlay, 2006). Ethics approval was gained from Queensland University of Technology (ethics ID number 3695).

## FINDINGS

Thirteen interviews were conducted virtually between 7 January 2021 and 19 February 2021 using video-conferencing software. Ten participants were working for refugee-specific service delivery agencies, including those that provide resettlement and health support ranging from general through to specialist care. Five participants worked in organizations that provide low- or no-cost physical activity programs within Brisbane, including those who provide community services government and a range of not-for-profit organizations that facilitate formal and informal exercise options. Several of the physical activity providers delivered programs specifically to refugee-background communities. Similarly, several refugee-specific service delivery agencies also provided physical activity programs. Consequently, there was an overlap in the knowledge and experiences shared by the two participant groups. All participants reported experience engaging with and delivering programs and services to refugee-background communities.

While it was not a participant selection criterion, several participants identified as members of refugee-background communities. Participants were also not asked to disclose specific personal demographic information such as age and gender. However, based on interviewer observations, all participants but one identified as women and ranged in age from young adult through to older adult, with most between the ages of 20 and 45 years. The role, experience and level within the organization varied for each participant. Several participants were senior leaders of not-for-profit organizations in Queensland; others were health professionals or managers of service delivery agencies. Experience also varied among participants, with younger participants indicating less experience and some older participants having more than 20 years of experience across community and health sectors.

### Improving cultural safety through intersectoral collaboration

All participants emphasized that, for participation among refugee-background communities in physical activity programs to increase, cultural safety and an intention to foster a sense of belonging need to underpin program delivery. However, participants expressed that currently, there is a lack of cultural safety within the mainstream sector that is restricting the accessibility and appropriateness of many programs for refugee-background communities. This was clearly articulated in descriptions of the ‘whiteness of [many of] the service[s]’ (P10) that deliver community-based physical activity programs and how this makes taking the ‘first steps’ (P3) toward participation highly daunting for refugee-background community members. Participants described the embodiment of whiteness in these spaces (see Fredericks, 2010) as the ‘unspoken barriers’ (P6) to participation in physical activity programs that leave members of refugee-background communities asking themselves questions like: *What if I don’t know how to fill out the forms? What if I don’t know what the person is saying?* (P1). Participants expressed that, where tailored and mainstream and formal and informal programs are used, a ‘blended’ or ‘staged’ approach to providing physical activity programs is needed to improve cultural safety and ensure that activities suit the different needs of different refugee-background communities. Several participants initially suggested providing supportive and culturally tailored physical activity options to refugee-background communities with the long-term view of creating a more welcoming and culturally diverse mainstream physical activity sector.

Relatedly, participants described the need for stakeholders, particularly sporting clubs, to take responsibility for the cultures of their own organizations to ensure that internal structures and systems foster equity and inclusion.

If we can make mainstream services more accessible and more inclusive and more culturally relevant to people, that is, much better and more sustainable, and it actually builds a more cohesive society. But it means you do need to modify what you are doing sometimes and make things genuinely accessible for people (P8).

Participants proposed various strategies, including diversifying the players on sporting teams, ensuring that staff within organizations were representative of diverse cultural backgrounds, upskilling volunteers and critically examining internal policies and procedures around diversity, inclusion and racial discrimination. Participants also spoke about using appropriate language and terminology, securing a practical time and appropriate venue, selecting activities that communities enjoyed, facilitating informal activities such as gardening or walking, ensuring costs remained low or preferably free, including the whole family or providing children’s activities and care, ensuring that participants could access culturally appropriate and comfortable clothing or swimwear, having sensitive and patient facilitators, providing gender-specific activities and providing an interpreter alongside the facilitator. Several participants discussed the value of bilingual consultants, who are typically respected community members with the capacity to speak multiple languages, for directing appropriate adaptations.

Participants considered improved collaboration and communication between stakeholders who deliver physical activity programs and provide services to refugee-background communities as a critical step towards improving cultural safety and program quality. Furthermore, participants expressed that sustainable partnerships between all the organizations that have a stake in refugee health and physical activity need to be developed to improve cultural safety and maximize the benefits arising from limited resources.

I just think the benefits of opening services up to different communities are great. The more that we connect with others and link others together to support one another—be it in our health services or sport or anything—the better the outcomes for refugee communities (P5).

Participants expressed that their respective organizations would benefit from a practical resource, such as an online directory or portal, which provided information on the availability of culturally safe community-based physical activity programs. Given their own resource constraints and heavy caseloads, several participants from refugee service delivery agencies emphasized the need for a lead organization or agency to take responsibility for coordinating physical activity for refugee-background communities.

### Confronting constraints imposed by the broader public policy environment

Participants explained that the organizations in which they work are fundamentally limited by a market-orientated program delivery model that increasingly renders low-cost physical activity programs unviable because ‘… there’s not a tonne of money in it … if this is their primary source of income, they want to get paid. They don’t want to do free work. There’s not much incentive to do it’ (P1). Participants who worked for small multicultural organizations deeply embedded within refugee-background communities that deliver grassroots physical activity initiatives talked about the difficulties in accessing government funding, particularly because tendering models privilege larger service providers.

One of the problems is you’ve got organizations who are actually embedded within the community and do things because that’s what the community needs, but then you’ve also got service providers who are looking for outcomes and are only doing the work to achieve those things because of the funding. They’re not complementary models … that’s why we’ve found the community-led stuff has the best value and has the best outcomes because they were doing it for themselves … The biggest frustration I have in this space is how organizations get funding … what actually ends up in the hands of the community or as a community outcome is not at all reflective of what that organization has got. I think a lot of funding models need to change. (P7)

According to the participants, the sector continues to rely on short-term grant funding cycles. Participants described a lack of dedicated, recurrent government investment that is causing programs to ‘fall over’. They reported that this inhibits ongoing participation and damages trust within communities. Here, it was explained that ‘the physical activity space … at the moment it is very ad hoc, very opportunistic – whenever we can, however we can and whenever we can, and the moment something changes, there is nothing’ (P3). These funding constraints were also reported to contribute to under-resourcing (staff, facilities, equipment) at organizational levels, which was seen to compromise program quality. Participants expressed that reporting requirements, especially those associated with funding, such as achieving key performance indicators, compromised the purpose and quality of program delivery to refugee-background communities and limited their ability to ‘actually be culturally responsive’ (P4).

Participants reported a lack of genuine consultation by local councils, state sporting bodies and larger settlement agencies with refugee-background communities during the design and implementation of community-based physical activity programs. When engagement with multicultural communities did occur, participants perceived this consultation to be largely driven by the organization’s agenda to meet key performance indicators and secure future funding. For example, participants working in not-for-profit organizations delivering services to refugee-background communities saw ‘consultation’ from leading state sporting bodies as highly tokenistic:

… basically, they [a state sporting body] would ring us up on Harmony Day and say *can you bring some multicultural people along? We’ve planned this event without any engagement, and now we don’t have any diverse people. …* They just make absolutely no effort whatsoever to connect with those communities unless it was Harmony Day or Multicultural Round, and that ticks their diversity box for the year and got them their KPIs [key performance indicators]. (P7)

Relatedly, the current policy-making environment was described as ‘very bureaucratic’ (P9) and a lack of political will at local and state government levels to redress inequitable physical activity participation among refugee-background communities was articulated. The current policy approach was likened to ‘… just putting a bandaid over the big problem, which is very much the policy invisibility and lack of investment and strategies around these issues [refugee health]’ (P4). Participant 4 expressed that the issues articulated during the interview were ‘the symptom of the biggest issue, which is the inequity issue in Australia when it comes to investing in the health and wellbeing of all of our populations’. Another participant expressed the need for a roundtable discussion with government and key stakeholders and stronger political leadership to develop a strategy to address the structural and systemic barriers that refugee-background communities encounter in Australian society. Currently, as the participant’s narrative below suggests, while it can be assumed that approaches to cultural diversity have become more progressive over time, this may not be the case:

At the macro level, the CALD [culturally and linguistically diverse] population is seen as the population that should fit with the mainstream … And if you are creating everything on that presumption, of course, you don’t want to have any special dedicated strategies—most of the time if you even have them, they are just tokenistic. And if you go the next step, where there is an action plan or investment, there is nothing, so it’s quite clearly just a token … At the end of the day, it’s actually discrimination because there is still expectation from mainstream administration that migrants of non-English speaking background should fit or assimilate or integrate into the mainstream culture. They may sound outdated, but that still is the reality. (P4)

### Building capacity and empowering the community to diversify the sector

Participants expressed that building the capacity of refugee-background communities to deliver community-based physical activity programs is an important means by which the sector could diversify and participation rates increase. Participants shared various ways this capacity could be built, including government investment in the informal physical activities in which members of refugee-background communities already engage. For example, several participants shared stories of groups of men from refugee-background communities who play football together socially on local fields, as well as a young man of refugee background who delivers low-cost personal training sessions to refugee-background mothers.

Relatedly, participants expressed that there is great potential and scope to increase workforce diversity by providing members of refugee-background communities training and employment opportunities within the community-based physical activity sector. Participants described how this could be achieved through targeted employment positions, internship programs, or the provision of coach and referee accreditation courses to members of refugee-background communities. Several participants suggested that an increased number of bilingual professionals within physical activity organizations would contribute significantly to breaking down cultural barriers. In this context, bicultural workers were described as ‘community connectors … who can be a bridge and bring in engagement’ (P11).

Participants also identified opportunities to upskill refugee-background communities in the areas of program design, risk management and business development. It was expressed that these investments empower refugee-background communities to drive physical activity programs themselves. Participants viewed capacity-building approaches that engage refugee-background communities in culturally safe programs, provide employment opportunities and enable fuller participation in community life as more effective and sustainable than the current status quo. However, proper leadership and investment were considered critical for this to be realized.

We are ignoring the social determinants of health, and we just sort of come up with these mainstream, middle-class strategies and try and retrofit them by putting it in more colourful brochures and other languages … It’s much more important to look at the people not as migrants and asylum seekers and refugees, but to look at their cultural background, their gender, their socioeconomic status, their education background, where they live. They are actually the parameters you should look at if you would like to engage these people in the physical activity … We can tinker all we like downstream, but unless we play upstream, we are not going to make any lasting impact. We can come up with some great models that work at the grassroots, but ultimately, who is going to be the steward of that—who owns it, who is going to invest in it? (P4)

## DISCUSSION

Participation in physical activity initiatives provides important opportunities for members of refugee-background communities to regain or maintain good physical and mental health in a different socio-cultural environment and form a sensation of connection to and belonging in place (Spaaij, 2012; Spaaij, 2015; Mohammadi, 2019; Malatzky *et al.*, 2020b). This participation also has recognized benefits for members of the broader community who belong to dominant cultural groups (McLaren, 2003; Dandy and Pe-Pua, 2015). This is emphasized in human geography scholarship, where the ‘coming together’ of ethnically and culturally different others to exercise has been found to facilitate social intimacy and enhance feelings of connectivity between culturally different individuals (Neal *et al*., 2015).

Refugee-background communities are often understood as ‘hard to reach’ within dominant public policy discourses (Brackertz, 2007; Brackertz and Meredyth, 2009). However, from the participants’ perspectives, serious discussion about why these communities may be hard to reach and how this could be overcome at a local community level remains marginal. Participants in this study provided rich insight into the deep structural and systemic inequities that continue to marginalize and exclude refugee-background communities from participation in community-based physical activity programs. It is important to consider the significance of these barriers in the context of contemporary findings that participation in such programs plays a key role in democratizing cities and facilitating broader civic participation and can thus be understood as a fundamental right to and in place (Aquino *et al*., 2022).

Participants articulated a lack of culturally safe and appropriate programs and emphasized the inequitable access to the social, economic and cultural resources needed for participation within refugee-background communities. The findings of this research suggest that localized, intersectoral collaboration—where there is a genuine partnership and established communication channels between service providers of physical activity programs and those working specifically with refugee-background communities—is central to improving the accessibility of, and thus participation in, mainstream community-based physical activity programs. While the focus here has been on participation among refugee-background communities, the approach to bridging the gap between programs on offer and community needs described and advocated for by participants could be more broadly applied to engage many other ‘hard to reach’ population groups (Brackertz and Meredyth, 2009; Malatzky *et al.*, 2018).

Relatedly, the approaches recommended by participants in this study recognize the importance of not reifying members of refugee-background communities based on this status. Other aspects of social identity or social location, including gender, age and socioeconomic status, were recognized as equally relevant (Balram *et al*., 2022). Inclusive approaches to community-based physical activity programs must also be intersectional and understand that people from refugee-background communities are not a homogenous group, nor are they defined solely by their refugee experience (Spaaij *et al*., 2019; Peers *et al*., 2023). Importantly, this research identified current barriers to realizing the kind of intersectoral collaboration that would enable such inclusive approaches to designing and delivering physical activity programs at the community level.

Participants described how the ability of service providers across related sectors to collaborate and deliver culturally appropriate and affordable physical activity programs to broad publics, including refugee-background communities, is constrained by the wider public policy environment. Participants explained that various physical activity programs exist at a community level. Not-for-profit organizations, health services, sporting clubs and local councils provide these at minimal or no cost. However, consistent with the findings of others (Russell *et al*., 2013; Ho *et al*., 2019), participants described these programs as *ad hoc* and unsustainable and confirmed that they were typically utilized by those who are already well resourced to navigate the broader healthcare system with efficacy.

These findings support the case for strengthening collaboration across the policy landscape, including Sport and Recreation departments, which hold a leadership role in the space of delivering physical activity programs (State of Queensland, 2023), as well as the broader health and social care system, to reduce fragmentation within the sector and improve population health (Cyril *et al*., 2016; Hartley *et al*., 2017; Ho *et al*., 2019; Mohammadi, 2019). Currently, participants in this study reported limited awareness about and communication between stakeholders across these sectors, which is maintaining inequitable access, service gaps and program duplication. It was evident that enhancing the accessibility of existing community-based physical activity programs requires considerable consultation and co-design with refugee-background communities. However, as participants in this study articulated, engagement in these processes must be genuine and ongoing rather than driven by political cycles and bureaucratic targets (Lenihan and Briggs, 2011; Larkin *et al.*, 2015; Sharmil *et al.*, 2021).

This study found that a key constraint on intersectoral collaboration and co-design with communities is the business, market-orientated model that currently underpins the broader health system (Baum *et al.*, 2016; Townsend *et al.*, 2020) and consequently, the provision of physical activity programs at the local community level. The current ‘top down’ policy approach to program design and implementation described by participants, which directs most funding to larger, mainstream organizations and settlement services rather than community, grassroots-level initiatives, is a poignant demonstration of this constraint. Relatedly, as participants described, grant and tendering processes are inconducive to establishing sustainable intersectoral partnerships, committing to community capacity building and enacting many practical strategies for improving cultural safety (Baum *et al*., 2016).

Participants articulated the implicit inequities embedded within these funding models and related government strategies. Their accounts demonstrate that the current ‘top-down’ policy approach fails to account for the multiple layers of disadvantage and exclusion that refugee-background communities experience across the social determinants of health. Consequently, intersectoral collaboration and the development of culturally safe and appropriate community-based physical activity programs are undermined.

It was clear from participants’ accounts that governments must move away from approaches that retrofit mainstream strategies to multicultural communities. As one participant expressed, ‘the programs that are most successful are the ones that are co-designed from the beginning—don’t just make something and come back to the community with it. Include their voice and include their participation from the beginning’ (P9). Participants’ collective desire was to rethink how refugee health is addressed within the community and public health sectors. Participants expressed that the sustainable and just course of action was to place a commitment to achieving health equity at the centre of the policy response and to privilege the strength and resourcefulness of refugee-background communities in developing community-based physical activity programs. The analysis highlighted the importance of place-based engagement in this process. Co-design and collaborative, community-generated strategies are built on ‘efforts and experiences in a given place’, and success depends on the participation of actors located within the ‘concrete context’ (Lam *et al.*, 2020).

The findings of this research indicate that shifting funding to communities and those organizations most engaged with the needs of communities is urgently needed. To increase refugee-background community engagement in community-based physical activity programs, these communities must be listened to and equipped with the resources and skills required to develop community-owned programs ‘from the ground up’. According to participants in this study, the most sustainable investment for local governments is in refugee-background communities themselves—building capacity to facilitate culturally safe physical activity within their own communities. Opportunities, training and support for providers who wish to deliver affordable, culturally inclusive physical activity programs to offer communities greater choice were identified as essential ingredients. Adopting ‘from the ground up’ approaches that engage with a diverse public and utilize the strengths and capacities of groups, whose disenfranchisement is often reinforced through deficit discourses, is an empowering way forward from the recurring debate around providing tailored ‘culturally and linguistically diverse specific’ programs versus mainstream programs that are more culturally inclusive and ‘diverse’. As the analysis here suggests, it is often a false choice.

Structural-level inequities and discrimination remain fundamental public health issues. Participants in this study expressed considerable frustration about the apparent lack of political will and commitment to funding support for multicultural health, including in the areas of physical activity and chronic disease prevention. The differing (and often conflicting) priorities of state and federal governments, local councils, sporting clubs and community organizations were identified as considerable challenges for stakeholders, yet many organizations in these sectors are well positioned to identify the policy changes and localized strategies required to increase participation in community-based physical activity programs.

## CONCLUSION

This research offers some key insights for enabling service providers within the community health sectors to bridge the gap between the health and social care needs of refugee-background communities and existing physical activity programs at a local community level. It is recommended that future research involve documenting a process of and lessons learned through forming sustainable intersectoral collaborations between the sector and community stakeholders that could be scalable and applied in other similar settings. Such processes hold great potential for empowering local communities to address the implications of broader systems that limit the participation of some populations within broader society.
